# Illusions of Understanding in the Sciences

**DOI:** 10.1007/s42113-026-00271-1

**Published:** 2026-04-30

**Authors:** Richard Shiffrin, Stephen Stigler, Frank Keil

**Affiliations:** 1 Indiana University, Bloomington, IN USA; 2 University of Chicago, Chicago, IL USA; 3 Yale University, New Haven, CT USA

**Keywords:** Understanding, Explanation, Comprehension, Deduction, Induction, Illusions

## Abstract

Scientists seek to understand the causes of observed phenomena. Beliefs that they have succeeded are based on understanding that is rarely or possibly never complete, and varies in depth and quality. Most often scientists believe they understand more than they do, making their belief an illusion. This illusion then persists in explanations scientists provide in print, in talks, or in discussions. The illusion that a scientist has a valid and complete explanation tends to be magnified when the data are well described by mathematical and computer simulation models due to the precision of such models and their ability to predict well; prediction does not imply causality, but gives the illusion that it does. The first part of this essay supports the case for the universality of partial and incomplete levels of understanding by showing the difficulty of reaching a deep level of understanding for even a simple analysis and model that most scientists use and believe they understand: linear regression. The second part highlights some implications of the existence of many levels of understanding and explanation, and their use by scientists for design, testing, analysis, and theory development. It discusses the way that deduction and induction depend on the levels of understanding and the implications of the illusion that a scientist’s understanding is deep. It makes a case that the many incomplete levels of understanding affect, often unwittingly, the ways scientists design experiments, test theories, comprehend, communicate, and teach.

## Illusions of Understanding

### Preface

Humans try to understand our universe in terms of causes that are often inferred from senses of agency and intervention: We push on a door and it opens. In most simple cases the result of an intervention is predictable. The first steps in forming such understandings in science are observations of regularities, relations that are often expressed as correlations and sometimes stated as ‘laws’. Starting with such observations, scientists form explanations through a process of induction^1^. They express their understanding of the causes of the data with theories. These are sometimes stated as verbal hypotheses of proposed mechanisms and sometimes as mathematical or computer simulation models that increase precision of explanation and allow quantitative prediction. As science progresses, the theories become more powerful and more valid, often doing so by explaining a narrow subdomain of phenomena, and more rarely, but importantly, generalizing and explaining a wider range of results.

Understandings are sometimes implicit and sometimes visual in character, but most often expressed with language, either in print or in explanations, and thereby inherit ambiguities of meaning. Such ambiguity of meaning applies as well to the terminology in this essay, terms such as “understanding”, “explanation”, “causality”, “deduction”, and “induction”. These are human conceptual constructs and will be understood and used differently by different readers. As such they resist precise definition and attempts to impose such definitions will limit the understandings and explanations reached by different readers. Our usage is therefore intended to be vague and admit of different connotations and meanings, and intended to match common parlance. Thus, roughly, understanding refers to an individual’s account of the causes of some phenomenon, explanation refers to the way an individual conveys their understanding to others, causality refers to the mechanisms thought to be most important in producing some phenomenon, deduction refers to the use of agreed upon rules of logic and mathematics to reach conclusions based upon agreed upon premises (though different deductions occur when there is disagreement about the rules and premises), and induction refers to forming a currently best causal account of some phenomena based on analyses of the data, past results and theories, and everything else known to the person carrying out the induction. That ambiguities of language abound in scientific practice is clear from fierce debates that often arise among researchers and theorists about the meaning and implications of experiments when those disagreements arise from different interpretations of the language used.

Understandings may produce particularly strong illusions when they are satisfying, even when scientists are aware the explanations are incomplete. Some scientists may be motivated by a plausible and satisfying explanation not to look deeper. Not looking deeper is natural if the incomplete theory is useful (C.S. Peirce, 1992/1898), and can be motivated by an awareness that all models are incomplete. George Box reprised a long scientific history in his 1976 publication with the phrase: All models are wrong, but some are useful (Box, 1976**).** Incomplete and partial causal accounts are indeed useful: they have led to the many advances in science that have benefited modern society. However, such incomplete accounts can also be deceptive, misleading, and/or wrong, and produce costs, such as slowing scientific progress, and even harm, as when poor science and a bad explanation led to erroneous conclusions that vaccines produced autism (Madsen et al., 2002).

It is easy to find multiple levels of explanations for almost any phenomena of interest to scientists, and those different levels might be useful for different purposes, or harmful for other purposes. Thus we may believe that humans are causing climate change by burning fossil fuel, a very shallow explanation of a very complex process, but useful if it leads to development of other forms of energy production. Conversely, taking the complexities into account could be and has been used to dismiss entirely the possibility of human agency. To take another example, a scientist may believe and publish studies claiming to show that forgetting occurs because humans inhibit and suppress unwanted memories. This is surely a shallow account (possibly a wrong one: Jonker et al., 2013; Deferme, Otgaar, Dodier, Körner, Mangiulli, Merckelbach, Sauerland, Panzavolta & Loftus, 2024; Raaijmakers & Jakab, 2013), but that belief may lead to useful empirical studies. Conversely, belief in this account could be harmful if incorrect, possibly leading to clinical treatments of adults presumed to have forgotten traumatic events in childhood when those events did not occur (Loftus, 1993; Loftus & Ketchum, 1994; Loftus, 1996).

Theories stated with mathematical equations or computer simulations add precision to theory, are often easily falsifiable, and are useful when they predict well. On the other hand, accurate prediction does not guarantee that even the producer of the model understands the way the model works. Examples of such failures to understand abound in the history of science. An early example involving a great scientist is described by Stigler (2006): In the 1690 s Isaac Newton was asked to assess which of three dice outcomes was the most probable. He correctly carried out the needed calculations and produced the correct answer. However, when offering a verbal explanation why the answer was correct, he missed a true explanation. His explanation, when applied to slight variants of the original problem, would have produced wrong answers. Thus accurate prediction can be misleading to scientists generally to the extent that it conveys the impression that the model explains well. This is especially true of mathematically specified theories that predict well (e.g., quantum field theory), and of some simulation models that often predict well (e.g. ChatGPT), but are enormously complex and difficult to understand by almost everyone.

Illusions of understanding can take several (overlapping) forms. Some that are commonly encountered are: (1) Illusions of explanatory depth (we think we personally understand things in more detail than we do). (2) Illusions of explanatory completeness (even if we don’t think we fully understand it ourselves, we think the best experts do). (3) Illusions resulting from understanding something other than the goal (e.g. we believe we understand the formation of memories because we understand the anatomy of the brain site, the hippocampus, that is needed for such learning). (4) Illusions due to simple statements giving a feeling of insight (such as when tautological statements seem insightful because they are framed in a reductionist manner). (5) Illusions (as described earlier) that one understands the cause of phenomena because there exists a model or procedure that predicts well. (6) Illusions of causal strength (attending to an observed relation makes one believe the causal connection is stronger than it is). (7) illusions that one can describe causes simply. (8) Illusions by the explainer that the recipient understands what the communicator intends. (9) Illusions by the recipient of an explanation that the communicator understands well and that the explanation is correct and complete.

Such illusions are inevitable in a universe that is infinitely complex, or almost so. Thus, all our theories (our causal explanations) are approximations. Giorgio Parisi, 2021 Nobel laureate in physics, said: “The truths of science are provisional, but not relative. Scientific truths are always ‘approximations to the truth’.” Many physicists are pleased that 150 years of experiments and theory development have produced quantum field theory, a theory that predicts observations of fundamental particles and their interactions incredibly well. Other physicists ask why this theory works, why the many constants have the values they do, why the processes interact the way they do, why some processes have symmetries and others do not. If reality is for all practical purposes infinitely complex, then all theories and understandings are incomplete. Thus not just novices but leading scientists are forced to use a variety of partial levels of understanding and explanation.

Even what seem to be simple questions asked about some observed events can lead to an unending and ever deeper series of questions. “Why do we see through glass and not other things?” “How does a mirror reflect light?” (Even physicists would struggle answering such questions at the deepest current level of understanding). This is often the case for queries from children, an example being a question asked of her mother by the first author’s five year old granddaughter: “I know how I was born and I know how you were born…but how was the first person born?” How would one start producing a deep answer and explanation? Would one start with the ‘big bang’? It is most likely that every question is fundamentally this complex: “What is water?” has a superficial answer much like a definition: “molecules with two hydrogen and one oxygen atom”. But asking what are molecules and what are atoms and what causes water molecules to form a solid, fluid, or gas leads to an unending series of other explanations.

History suggests there will always be better and deeper theories around the corner. Scientists working on the same problem often generate different types of explanations, and it is not always clear which level is ‘deeper’. Consider four scientists seeking an explanation of how monarch butterflies navigate thousands of miles to the same precise location while taking four generations to make a complete trip. Scientist 1 wants to understand functionally, explaining why it is necessary to have multiple generations because of a combination of lifespan limits and bioenergetics; Scientist 2 seeks to explain what navigational cues could provide such precision; Scientist 3 seeks to understand how location information is transmitted across generations; and Scientist 4 wants to understand the molecular basis of the navigation signals. Each of these offers an incomplete account, leaving gaps and the possibility of distortion. These four instances hardly exhaust the possibilities. In this case, and with many others, it is not obvious how to meld together the different types of explanations.

We argue that a variety of degrees of understanding and explanation play a critical role in every aspect of scientific practice and every type of theory construction, that different degrees of understanding exist for all people and all scientists, and that even the authors of a model vary in these ways. Consider, for example, scientists who make mathematical models for airplane wing design. The models work quite well, but even the scientists who develop the models admit they do not understand them well enough to explain them (Regis, 2020). Another example is bicycle stability; in this case the mathematics are not so clearly laid out because much of bicycle design has proceeded by trial and error, but both riders and designers are at a loss in explaining deeply the principles at work (Borell, 2016). In these examples, the scientists know and admit their inability to explain. Whether these scientists believe they understand fully is less clear. For example, some experts responding to the Regis article said scientists do understand airplane wing design and pointed to the (quite old) field of fluid dynamics and the Navier-Stokes equations. The Navier-Stokes equations are seemingly simple sets of partial differential equations, yet quickly become immensely complex as they attempt to integrate energy transfer across the different levels of scale intrinsic to any case of real world turbulence (Zhou, 2021; Eyink, 2024). The existence of these equations is hardly a demonstration of deep understanding. We note that airplane manufacturers and boat-hull designers are unwilling to rely solely on the equations; they test designs in wind tunnels and tank tests before incorporating them.

Many different levels of understanding and explanation are necessary and useful in science partly because they are used for different purposes: to pursue science well, to collaborate on a scientific study, to publish successfully, to communicate science to other scientists in fields of various distances from one’s own, to teach at different levels of education, to educate the public, to convince others of the validity of one’s understanding, to produce applications useful for oneself or society, and possibly just to give the generator or recipient of the explanation a sense that an explanation is satisfactory.

Partial understanding can take several forms. Sometimes orderly relations can be uncovered but not explainable by current theory, and a good causal account awaits (‘dark matter’ and ‘dark energy’ might fall into the category of attempts at explanation aimed to predict but many find unsatisfying). In other cases a causal account works extremely well in most situations encountered (giving rise to a belief that one has a correct causal account), but subsequent observations reveal the need for a refined model. An example everyone knows is Isaac Newton’s accounts of gravitational attraction from the 1680 s that awaited refinement by Einstein more than 200 years later. A truly deep understanding (perhaps one based on quantum gravity) is still being sought today.

It might seem that these issues only apply to complex situations like climate change, cancer, or the neural bases for cognition and action, or only apply to models such as quantum field theory or Large Language Models. We disagree and illustrate the pervasiveness of difficulties of understanding, illusions of understanding, and difficulties of explaining one’s understanding by discussing in detail in the next section of this essay a simple relation described by a simple model that most scientists know, use, and believe they understand: linear regression. This example is described at length because many readers may be surprised by its various complexities, and because it has been our experience that even researchers sophisticated in methodology and statistics believe they understand the issues better than they do in reality.

### “Simple” Linear Regression

**Fig. 1:**
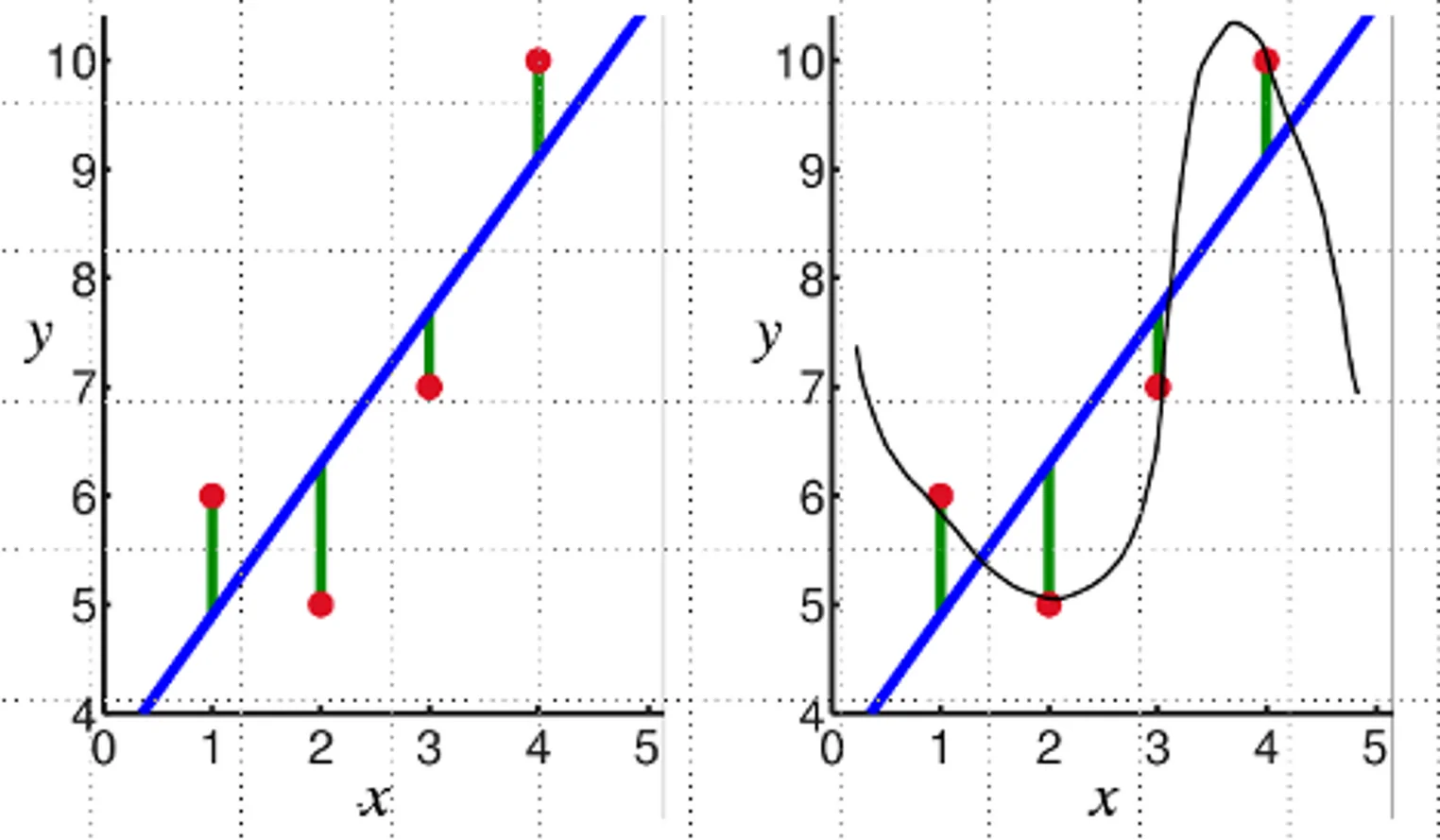
Left panel: The data are the red dots. The blue line, the linear regression, is a description of the relation based on assumptions that the data are noisy and describable as linear. To be precise, the linear model is described as y = ax + b + s, where b is the intercept at x = 0, a is the slope of the line, and s is the deviation of error assumed to be gaussian with zero mean and standard deviation sigma. The parameter values are commonly chosen to minimize the summed squared deviations of the observations from the predicted line. Right panel: The black line gives one of an infinite number of alternative descriptions, one that describes the data better, but at the cost of a more complex description, usually assumed to be ‘overfitting’. Assessing the validity of such alternative descriptions depends in good part on induction: What is seen as the best cause of the data

The data that are the basis for scientific theories is almost always in the form of a relation between two or more things we observe and measure, in planned studies or in the world. The simplest form of such a relation is the way that one measure varies in accord with another, often described as a correlation and shown as a scatterplot and a regression line. Thus, we might have the weight of a farmer’s steer on one axis and the market selling price on the other. The simplest relation between two measures and the one often used as a good approximate description of the relation is linear, as in the left panel of Fig. 1. In a perfect world the y values might lie exactly on a line, but there are always various types of measurement noise and the y values are only approximated by a line. What is noise depends on the sophistication and validity of one’s model: E.g. to take a non-linear example, deviations in planetary orbits from Newtonian predictions can be attributed to noise at one point in time, but ascribed to unseen other planetary bodies at another point in time, or ascribed to an improved model of gravitation at an even later point in time. Even when a best approximation is linear, noise in the data makes it possible to describe a relation with a complex form, as in the right panel of Fig. 1. To the extent that the proposed regression in either panel is a good one, then one can use it to predict well the expected value of y for a specified value of x. However, describing and predicting well are not the same as understanding the causes of the relation. Scientists interested in causes form a model of what causes the data pattern, a process of induction based in principle on the observed data and everything else they know.

The reader might well wonder why it is necessary to describe something so simple, something everyone knows. The primary reason is a common assumption that a linear description is a causal account, for example that the value of x causes the value of y, However, correlation does not imply causation (e.g. Hume, 1739). An example is from Simon Newcomb in 1886, who noted that a correlation of increased quinine consumption and increased fever should not have implied that quinine caused fever, but due to selection of people in regions with lots of fever taking quinine (Newcomb, 1886, p. 36). There are many examples where it is or becomes clear that two correlated factors are not primary causes of each other (e.g. smoking is traditionally correlated with alcoholism but neither is the primary cause of the other).

In some cases it is reasonable to conclude that one factor indeed is a primary cause of the other: E.g. If the dosage of a drug is observed to vary approximately linearly with the proportion of patients that recover. Of course the validity of that induction depends on a host of other assumptions. For example, if higher dosages were being given to sicker patients a different explanation might emerge. In general, the mere fact of attending to a relation between two factors tends to lead to an overestimate of the strength of causal connection between the two (likely because one tends to downgrade or ignore the importance of other causal factors; e.g. Gandhi et al., 2024), and because it is often difficult to imagine a third cause that is not provided. In general, the finding of ‘best’ causes is the subject of scientific induction, and is far from trivial.

The fact that linear regression is just one of an infinite number of descriptions of a given data set itself demands consideration of causes: Linearity is often assumed due to the principle of parsimony, Occam’s Razor, when coming up with a causal account. A preference for simpler explanations, everything else being equal, has been a popular principle well prior to Occam in the 1200’s: Aristotle (1991) writes in the 300 s BC in his *Posterior Analytics*, “We may assume the superiority *ceteris paribus* [other things being equal] of the demonstration which derives from fewer postulates or hypotheses” (for recent discussions of simplicity and competing biases, and cases where more complex models may be preferred, see Johnson et al., 2019; Lombrozo, 2007; Zemla et al., 2023). The phrase “other things being equal” covers a lot of territory: As science progresses the best accounts often grow more complex. E.g., when people first observed they could see through glass, they may have been satisfied with an ‘explanation’ that glass is transparent; the best account today emerges from quantum field theory and is far from simple. However, starting with an assumption that a relation can be described well as linear, there are still many difficulties of understanding, as covered next.

### Simpson’s Paradox

Recognition of the problems of understanding described by Simpson’s paradox (Simpson, 1951) and date back at least to Yule (1903), but that has not prevented misunderstandings and invalid conclusions persisting to the present day.

Many people know of Simpson’s through simple examples. One was UC Berkeley admissions showing that individual departments admitted more women than men but the university as a whole had admitted more men than women (Bickel et al., 1975). Another is due to the demographer Nathan Keyfitz, based on data from 1971 to 1976. He was examining the way that the number of children per family varied by Canadian province and by culture. The results shown in the first two columns of Table 1 seem to indicate that French-speaking Canadians are producing larger families, both in Quebec and other provinces. However, in all of Canada combined, the average numbers of children per family were larger for English speaking families, as shown in the last column of Table 1. The apparent contradiction is due to the fact that 85.3% of the French-speaking families were in Quebec, while only 6.1% of the English-speaking families were in Quebec. The overall results are due to a weighted average: French: 1.80(0.853) + 2.14(0.147) = 1.85; English: 1.64(0.061) + 1.97(0.939) = 1.95.

**Table 1:** Examples of Simpson’s paradox: The numbers given are children per family for the indicated group

Language Spoken in Home:	Quebec	Other Provinces	All of Canada
French	1.80	2.14	1.85
English	1.64	1.97	1.95

**Fig. 2:**
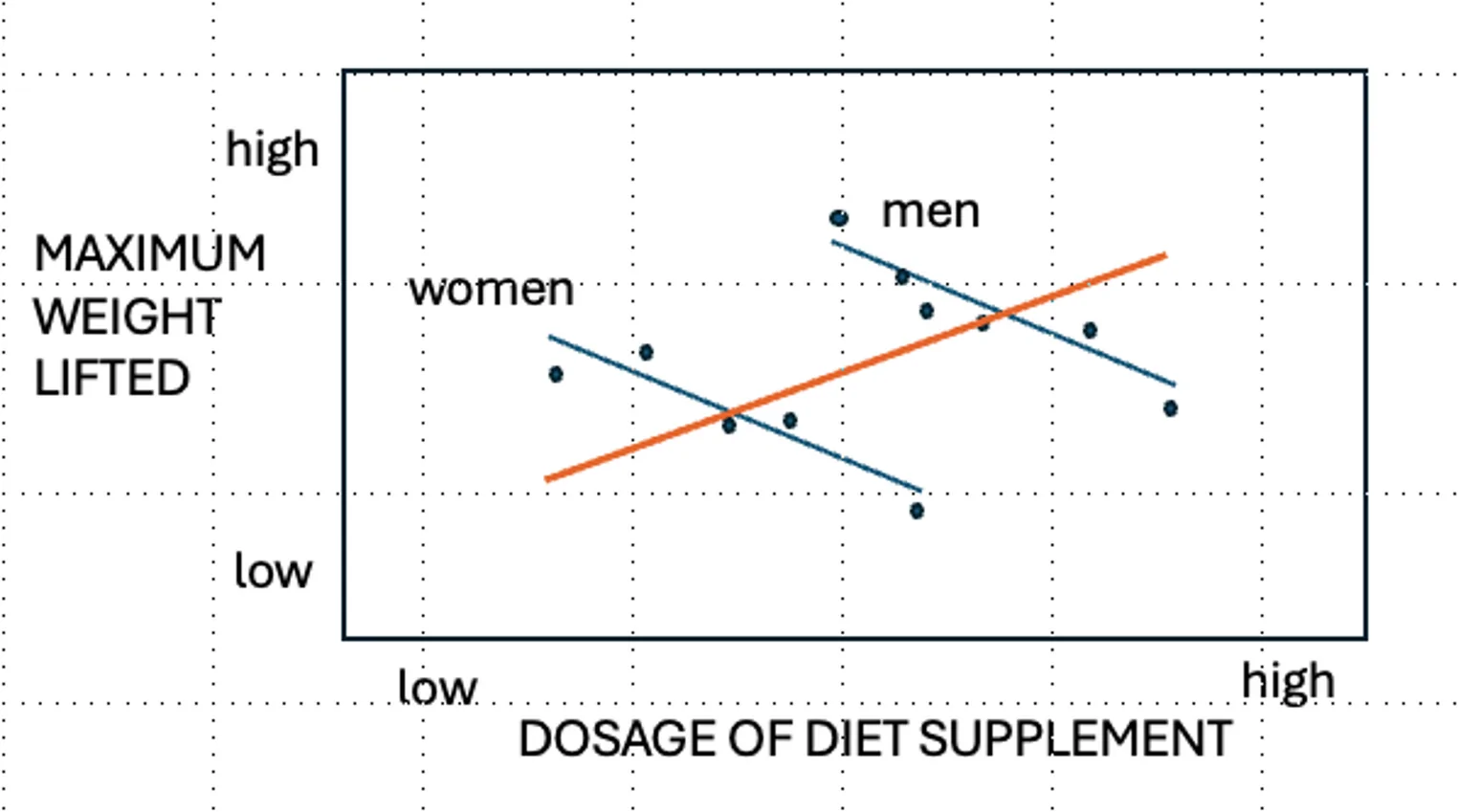
Ability to lift weight as a function of diet supplement dosage

Here we see subgroups showing one trend and the population showing the opposite trend, due to mixing of proportions with different probabilities. Most people find such situations difficult to grasp intuitively. Thus, to aid understanding, Simpson’s is often described graphically: In the hypothetical example of Fig. 2 we see that higher dosage of a diet pill reduce ability to lift weight, both for men and women, but in the population as a whole the higher dosage produces a weight gain (perhaps because men being heavier are given higher dosages).

**Fig. 3:**
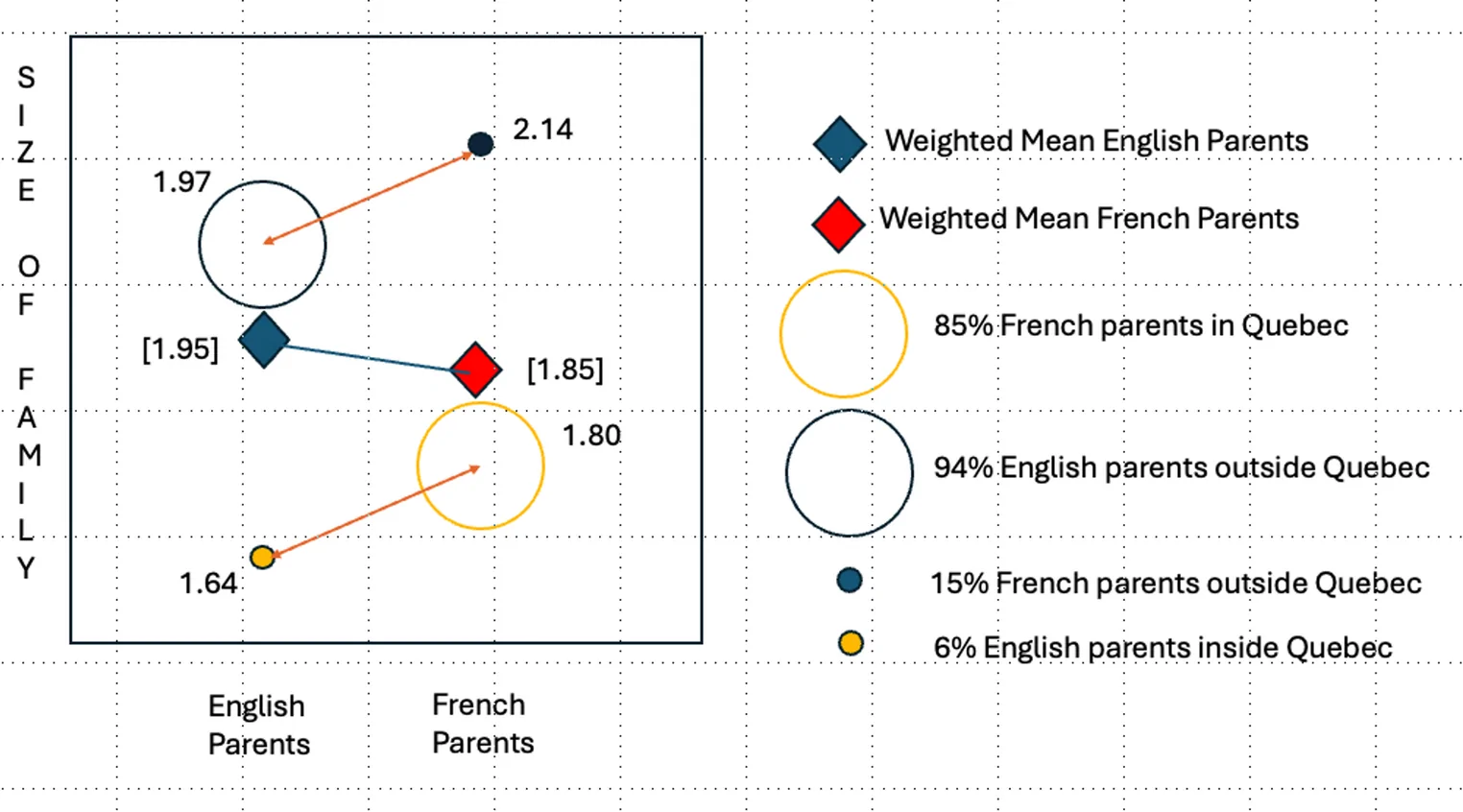
The Keyfitz example in graphical form. Circle size gives population size

A graphical depiction like that in Fig. 2 makes it easy to see how a global trend can be opposite to local trends. However the connection to the examples given earlier, based on mixing of proportions and probabilities is itself rather difficult to understand. There is a connection, of course, because these situations are both described as Simpson’s paradoxes. This is illustrated in Fig. 3.

There is a tendency to dismiss Simpson’s as a rare event. This is a mistake. It is a very common phenomenon. It can appear whenever data sets are combined. It can reverse the direction of an effect or strengthen it. Simpsons represents a confusion of overall trends (regressions) with conditional trends (regressions). Good and Mittal (1987) use the term “amalgamation paradox” for examples such as Simpson’s paradox. Invalid conclusions caused by this confusion have persisted to the present day, even for these relatively simple cases with just two variables. What is more, there is always the nagging feeling that an important “third” variable has been omitted from the analysis, a “lurking variable” in George Box’s evocative phrase (Box, 1984, p. 157). As difficult as it is to understand the role of Simpson’s in these simple cases, the difficulties rise enormously when the number of variables increase, or when the regressions for the groups are non-linear.

Simpson’s situations seem paradoxical in part due to a general difficulty humans have in probabilistic reasoning. This is shown for example in the work of Kahneman and Tversky (e.g. the ‘Linda’ problem showing readers judging a conjunction of two events to be more probable than one event: Tversky & Kahneman, 1983) and as shown by the difficulty many people at all levels of education and expertise have with Monty Hall problem (Selvin, 1975). Humans led to give quick intuitive answers about probabilistically defined problems often give wrong judgments even when they are experts in probability (e.g. Sadler-Smith, 2023).

Simpson’s paradox provides us our first hint that linear regression may be more difficult to understand than it appears.

### Regression to the Mean

Regression to the mean is a statistical phenomenon that occurs universally when two paired measures are not perfectly correlated. If a member of one pair of values is extreme (in either direction), its partner would be expected to be less extreme. For example, if the pairs are students’ scores on a midterm test and a final exam, an extremely high score on the midterm would (on average) be followed by a less extreme score on the final. It is not that the student has become less able, but the lack of perfect correlation implies that both scores are subject to transitory components, and large values of the transient components in the same direction would be unexpected. This description may not be perfectly explanatory, so it is worth unpacking it further.

**Fig. 4:**
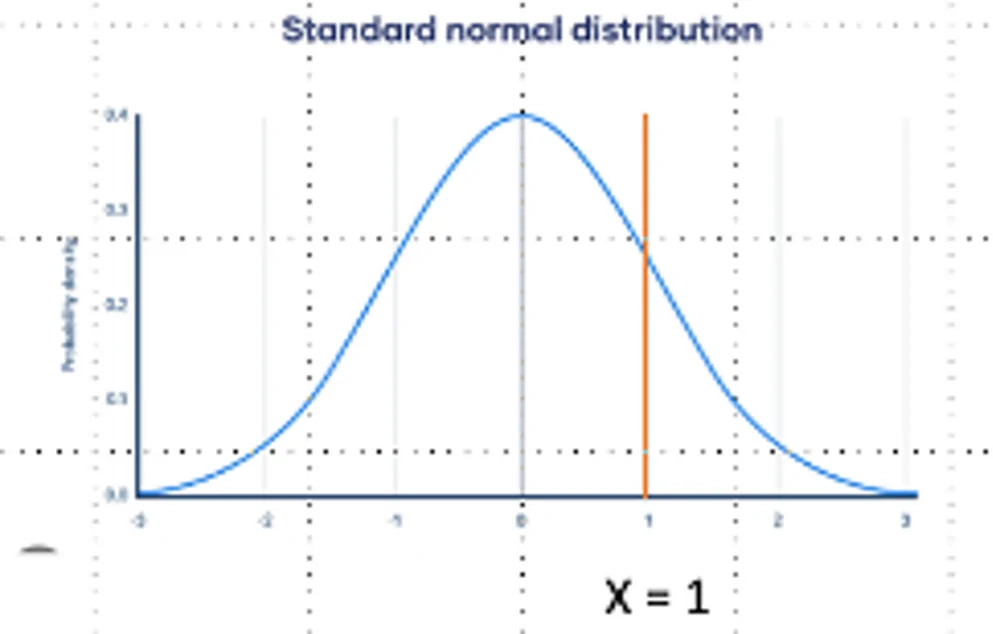
A sample is taken from a standard normal distribution

The simplest case, though not the one of interest, is easy to describe and explain, and is illustrated in Fig. 4. A sample number is taken from a standard normal distribution, and its value is indicated by the red line. If we sample again, independently, what value do we expect? Obviously we expect the sample value, on average, to be at the mean. In this simple case we see regression all the way to the mean: The observation x = 1 does not alter the expectation of x = 0 for the next observation. However, the interesting cases occur when (1) there is regression not to the mean of the source of the original observation, but to the mean of a group containing the member whose initial and final observations are of interest, and (2) when there is more than one group described with linear regressions.

The first case can be described with the following example: Let each individual in a group be measured imprecisely so that each measurement is a sample from a gaussian distribution (as in Fig. 4). The individual distributions have different means, but when a sample is taken for an individual, the mean is not known. What do we expect if we take another sample from that individual? This is no longer a trivial question. It was first addressed properly by Galton in the 1880s when he took a major step towards creating multivariate statistical analysis by analysis of a novel model he invented (perhaps built) called the Quincunx. It was like a pachinko game - small balls were dropped through a sequence of alternatingly offset rows of pins and their patterns recorded at different points in the fall. A full discussion can be found elsewhere (Stigler, 1986, 1999, 2010), but one simple aspect that led to regression toward the mean is easy to describe.

**Fig. 5:**
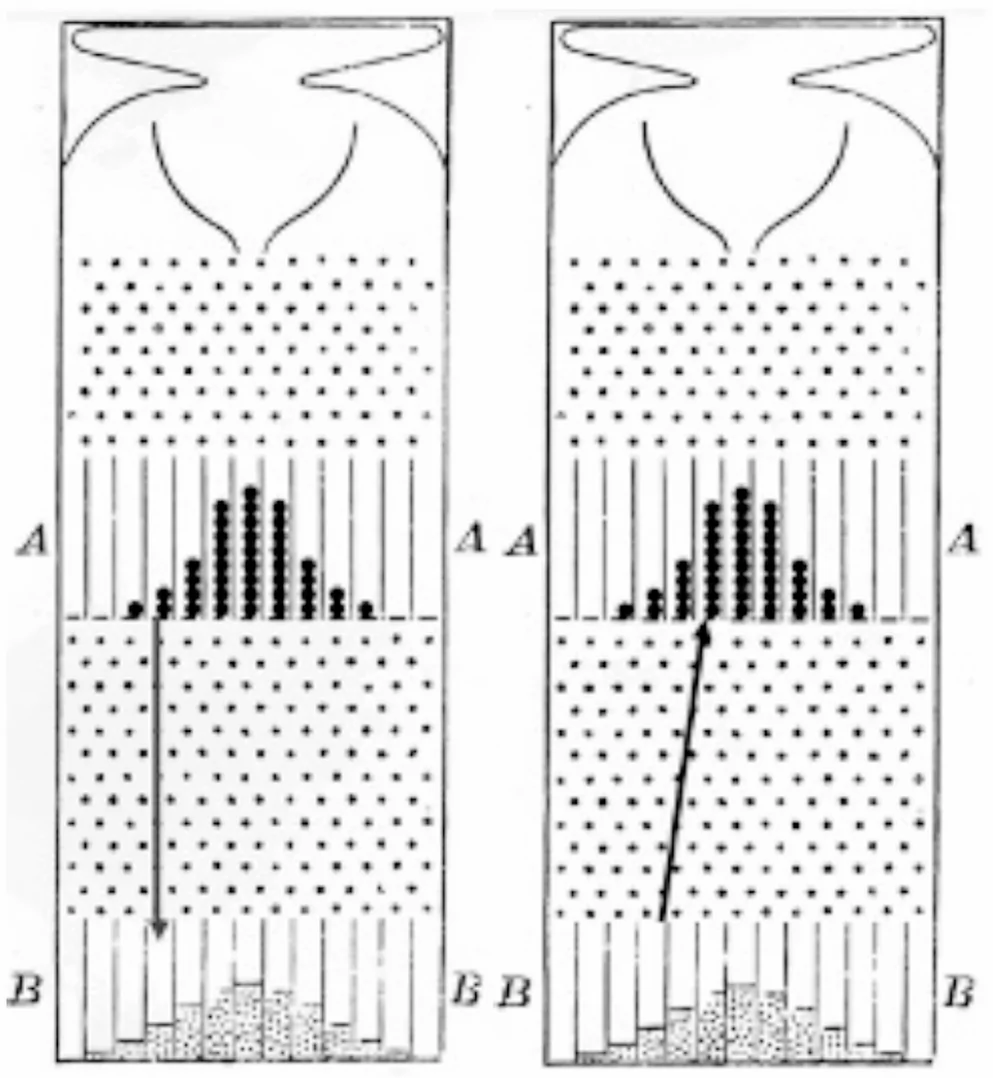
The ‘Quincunx’ built by Galton in the 1880s

Figure 5 shows a pattern like a gaussian distribution found if a number of balls are stopped at the A level; if then released to fall further, to the B level, the shape will again be like a gaussian distribution but more spread out. Now consider balls in the fourth compartment from the left on level A of the left panel: When released they will tumble down randomly, on average to just below where they start. Now look at the fourth compartment from the left at the B level and bottom of the right panel of the figure and ask: at the bottom of the right panel of the figure and ask: From which of the A level compartments were the most likely source of those balls? The most likely average source is nearer the center, illustrated by the arrow pointing to the right, for the simple reason that there were more in the center that could wander to the left than from the extreme left wandering to the right.

**Fig. 6:**
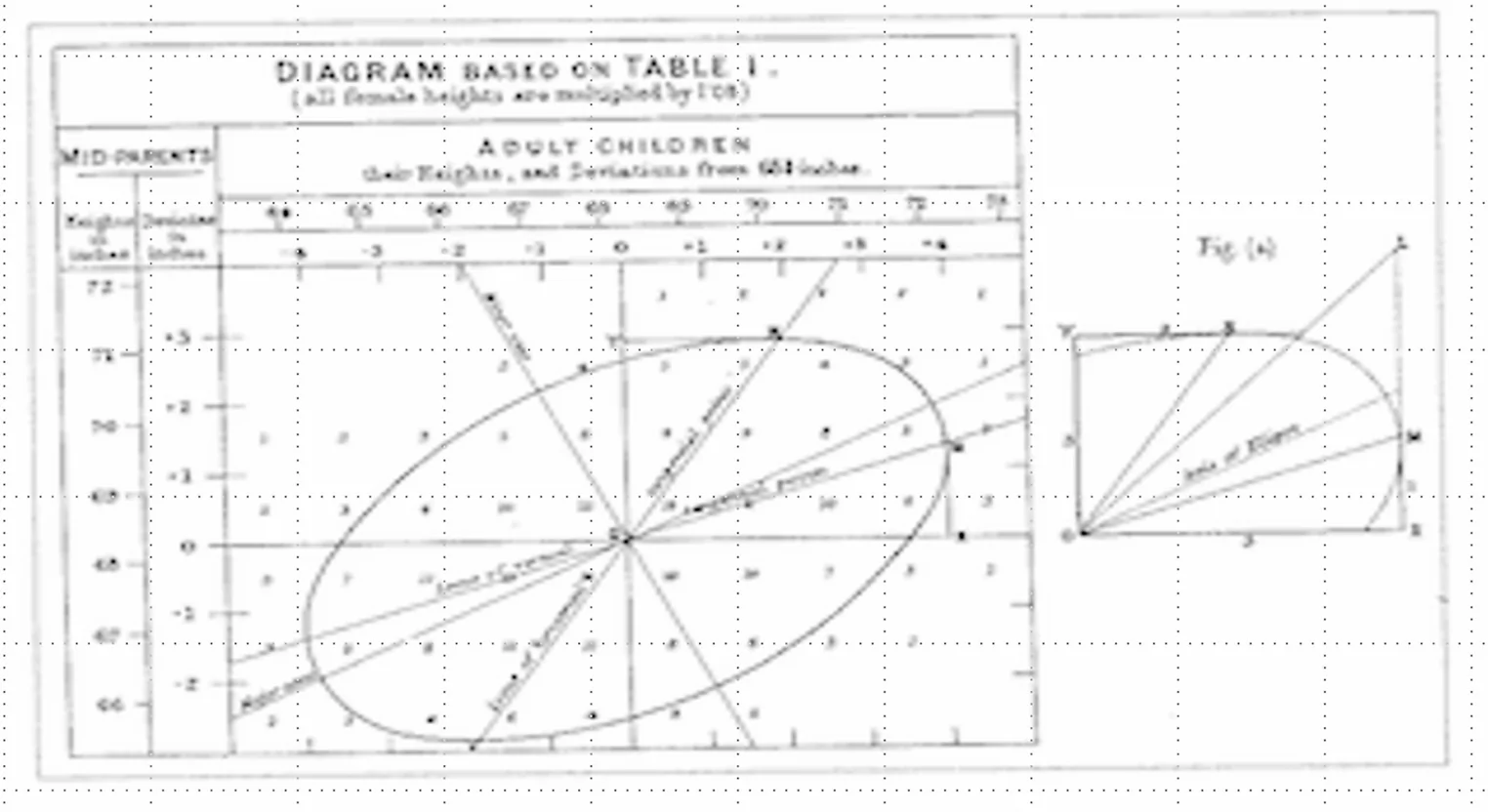
Galton’s analysis using linear regression to show how parents tend to be shorter than their adult children and adult children tend to be shorter than their parents

Galton was not interested in Pachinko games, but rather an explanation for puzzling data he collected showing that adult children of a given height tended to have parents shorter on average than them and parents of given average height tended to have children shorter than them. The explanation in both cases is regression to the mean. Thus, Galton explained how regression to the mean operates in a moderately complex real-world setting. Galton’s figure showing his analysis in terms of regression lines is given in Fig. 6. We will not try to unpack and explain this figure here, but a detailed explanation can be found in Stigler (2016).

Regression to the mean occurs in uncountable numbers of real world situations. Here we mention just one, known as the “Winner’s Curse” (Thaler, 1992). Of many examples here is one: Atlantic Richfield Co. (ARCO) sometimes bid in government auctions for the right to drill in various places. ARCO relied on experts to predict how much oil it would find, and therefore how much it should offer to pay. But when it won an auction, it usually found less oil than expected. Why? Because a winning bid always anticipates more oil than the average of all the other firms’ predictions, and that average tends to be more accurate (a ”Wisdom of Crowds” effect; Galton, 1907; Surowiecki, 2004). The more bidders, the more likely the winning bid is too high.

Although regression to the mean is a statistical phenomenon, the results are often interpreted causally and inappropriately so. For example, suppose one observed parents to be shorter than their adult children and proposed the cause to be a difference in diet. Indeed dietary differences might be a contributing factor, but the additional observation that adult children tend to be shorter than their parents would suggest the best explanation should be found elsewhere. Regression to the mean is often mentioned in statistics courses, but seldom in the detail needed to foster full understanding, so it is not surprising that, to the present day, scientists regularly produce causal accounts for data patterns that are produced by regression to the mean rather than causal mechanisms. Importantly, the examples we have presented and that Galton discussed are very simple in comparison to similar regression phenomena that can be expected to operate in more complex situations often faced by scientists, ones in which there are more than two variables that might be related in complex ways, and ones in which the relations are more complex than linear. In cases like that even the best scientists would have trouble reaching valid causal attributions. This occurs throughout the sciences in topics ranging from climate change, to the genetic instability of tumors, to protein folding, to human cognition, to the workings of economies.

### Lord’s Paradox

Understanding how regression to the mean works is not trivial, even for linear regression used to describe the relation of two variables for two groups. However, understanding the causes of the data that are described with linear regression is a matter of induction and inherently harder. This is demonstrated by Lord’s paradox.

**Fig. 7:**
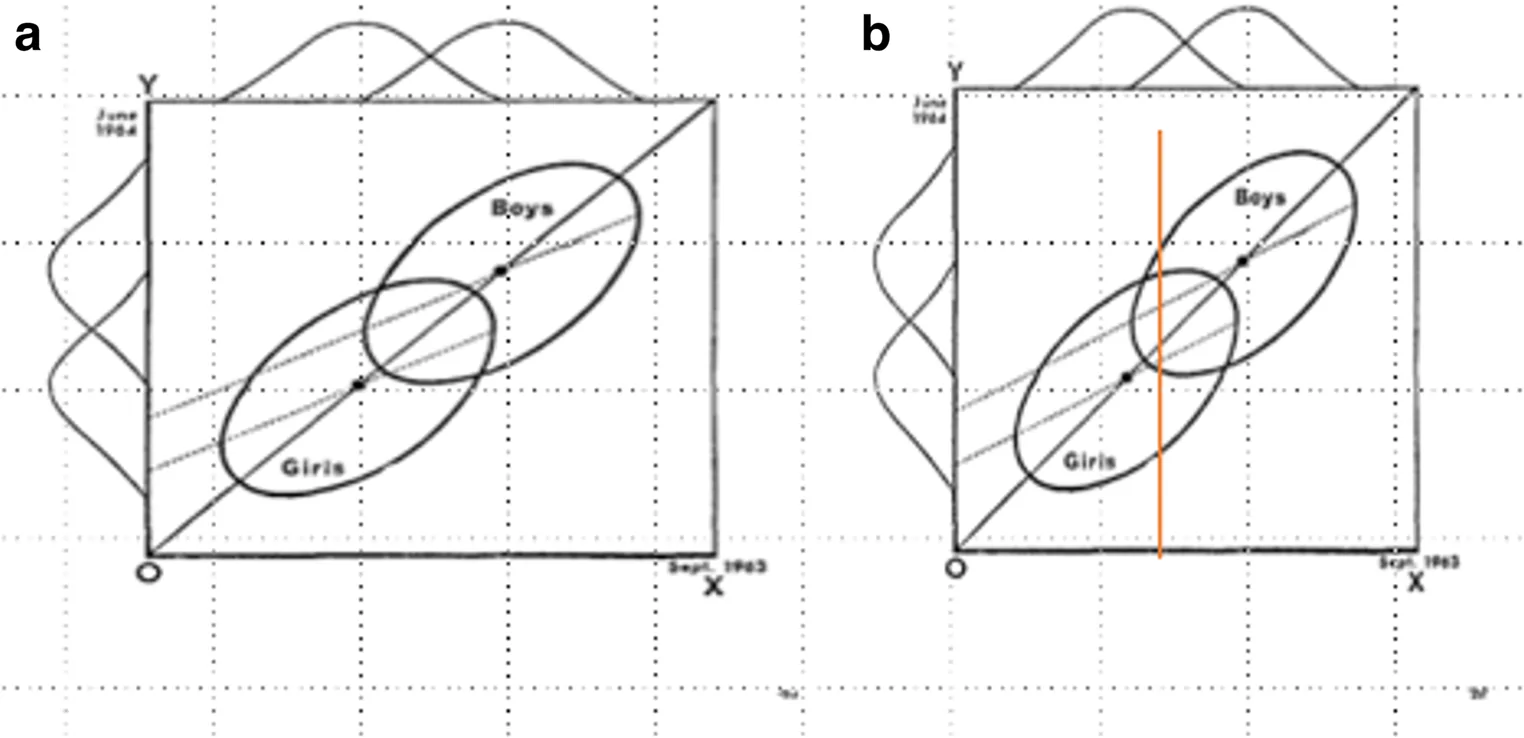
**a**: The figure from Lord (1967). **b**: A boy and girl of equal initial weight

In 1967 Fredric Lord, a statistician for the Educational Testing Service, published a two-page paper in Psychological Bulletin intended to warn researchers about proper use of Analysis of Covariance, a method of statistical analysis that permits making comparisons while “correcting” for some associated variable. It was titled: “A Paradox in the Interpretation of Group Comparisons”. The essence of the paper was given in its one figure, shown here as the Fig. 7a, the left panel.

In this hypothetical example^2^, boys and girls are weighed, first when they arrive at school at the beginning of the term, and then again at the end of the school year. In Lord’s data the *average weights* in each group did not change over the year, but *individuals’ weights* did change: some increased and some decreased, but on average there was no change in each group.On the horizontal axis are the initial weights, and on the vertical axis are the final weights. The two gaussian distributions of weights for the beginning of the year are given along the top of the diagram, and for the end of the year along the vertical axis, and are the same at both times for both boys and girls (albeit the boys are heavier). The ellipses in the center are meant to represent schematically what a scatterplot of the pairs (X, Y) = (before, after) for all the individuals would look like -for boys and girls they are each ellipses that are symmetrical around the 45 degree line (the line that represents individuals whose weights did not change). Neither group has any average change in weight during the year; individual’s weights did change.

Lord describes statistician 1 who concludes there is no evidence of any systematic effect of diet or anything else on student weight, and in particular no differential effect for the two sexes. We would hope that the readers, scientists or otherwise, would agree. However, Lord describes statistician 2 who analyzes the data “correcting” for sex, amounting to plotting a linear regression for each group, as shown by the two dotted lines with equal slopes. These lines can be used to predict the average later weight for a given initial girl’s or boy’s weight, by locating the initial weight on the X axis and then reading the heights above that value separately for the two lines. Statistician 2 notes the vertical difference in the two lines (the same difference above any initial weight) and concludes “… the boys showed significantly more gain in weight than the girls when proper allowance is made for the differences in initial weight between the two sexes”.

What statistician 2 missed was the phenomenon of regression to the mean. That effect is a selection effect. Suppose we compare a girl and boy of equal weight on arrival at school, indicated by the red line in Fig. 6b. At that initial weight, the predicted average final weight for a boy is lower, and the predicted average final weight for a girl is higher. Looking at an individual near that red line amounts to selecting a heavier than average girl and comparing her to a lighter than average boy. In the groups of both boys and girls, heavier than average members tend to lose weight and lighter than average members tend to gain weight.

It is hard to read the justification by statistician 2 without amusement, presumably what Lord intended. Lord presented the argument of statistician 2 thinking that the readers would see its absurdity. Perhaps because he titled the article a ‘paradox’, many readers did not get the point. He therefore published a 1.25 page paper in Psychological Bulletin two years later (Lord, 1969) pointing out that the conclusion of statistician 2 was ‘absurd’. More generally, Lord was making the point that a researcher should not blindly apply a statistical method without thinking about what the method was actually doing and what inferences were reasonable.

To many researchers (and us), Lord’s conclusions seem reasonable, but the difficulties of inducing the meaning of Lord’s data is illustrated by the many respected statisticians and causal theorists that have found reasons to support statistician 2. In the years since 1969 to the present day there have been a series of articles arguing variously that statistician 1 might have been wrong and statistician 2 might have been correct (e.g. Holland & Rubin, 1983; Wainer, 1991; Holland, 2005; Senn, 2006; Wainer & Brown, 2004, 2006; Arah, 2008; Pearl, 2016; Kim, 2018; Tennant et al., 2023). Some of these authors point to ambiguity in the causal question being asked. Others focus on the fact that a boy tends to gain more than a girl of equal weight (as shown in Fig. 7b). Are multiple interpretations of Lord’s data reasonable? One reason we find Lord’s take compelling is the symmetry of the data shown in Fig. 4a. One could plot the same data with end of year weight on the horizontal axis and initial weight on the vertical axis and the figure and its regression lines would be identical. The conclusion reached by statistician 2 would now be that boys have gained more weight than girls from the end of the year to the start. This would be the exactly opposite conclusion reached by analysis of the original way of plotting the same data. Some readers might find this example compelling, others not (also see Shiffrin, 2020). What can be stated definitively is that the interpretation of linear regression is far from trivial, and difficult enough to cause debate among leading researchers.

At this point, many readers may be coming to the realization that understanding linear regression is more difficult than first imagined. For those who remain unconvinced, we give one more example.

### Stein’s Paradox

As with Lord’s paradox and Simpson’s paradox, Stein’s paradox (Stein, 1956) is not a paradox, but a result that seems intuitively wrong. Suppose we take several noisy measurements (a sample) of some quantity; it is long known from many different perspectives that the best estimate of the true mean is the mean of the sample. Suppose we take such samples of three or more possibly unrelated quantities, perhaps a baseball player’s monthly batting averages, the yearly yields of a farm in Oregon, and the weights of Kindergarten children in Atlanta. The best estimate of each true mean is each sample average. However, Stein’s result (paradox) shows that a better estimate of all three means together requires shrinking the three sample means, either to their common mean (the Efron-Morris estimator) or toward zero (the James-Stein estimator). It seems preposterous that knowledge of the samples of unrelated quantities can help produce a better overall estimate (i.e. reduce the expected aggregate summed square errors from the three true means). Stein’s mathematical analysis is not in question, and the result is not hard to verify empirically (as was done by Efron and Morris in *Scientific American* (Efron & Morris, 1977); also see Efron & Morris, 1975). The phenomenon requires treating the problems in terms of a common criteria (the sum of the three separate sums of squared errors) so even if some of the three may lose accuracy, the overall criterion reigns in large errors, giving overall improvement. The phenomenon requires the squared error measure (so large errors are very costly), and the technical result requires normal error distributions. We mention Stein’s result because Stigler (1990) shows that the paradoxical nature of Stein’s result can be seen as a confusion of two different linear regression lines (as noted by Galton in the case of family stature).

### Multiple Ways of Understanding Linear Regression

We have seen that seemingly simple linear regression is difficult to understand, even for experts. The apparent simplicity of linear regression is partly due to its easier-to-understand use in predicting data, but its simplicity disguises many different ways to interpret the data and to understand what is causing the data. Here we list some of the many ways of understanding linear regression.

a *Describing a relation*: Perhaps the simplest and most superficial understanding is that the relation of two variables is approximately linear. This may be correct as far as it goes, but it bypasses deeper questions such as the nature of the noise in measurements, the error criterion used to establish the relation, the joint statistics of the relation (their covariation), and changes in the relation that depend on the grouping of the factors that are jointly measured.

i. *Understanding the relation of multiple groups that use the same variables, and understanding how to compare those groups.*
ii. *Understanding measurement noise.*
iii. *Understanding the joint statistics of the relation.*
iv. *Understanding the criterion used to justify linearity.*

b *Understanding the use of linear regression for prediction*
c *Understand the statistical basis for regression to the mean* (and other statistical factors that affect prediction).
d *Understanding the causes of Simpson’s paradox*, including data produced by mixtures of groups with different probabilities, and the different forms of regression that vary with local and more global descriptions.
e *Understanding the statistical basis for Stein’s paradox*.

And most difficult of all, understanding the causes of the data described with linear regression:

f *Understanding the differences between deduction and induction.*
g *Understanding which variables are most important: *In most situations encountered in the field or in the lab, there are a host of causes at least partly responsible for the data. Thus there is a general need to identify the most important causes. Informally, scientists often freely acknowledge the existence of additional causes but also relegate them to a category of “external” influences, implicitly downgrading their importance. As one example, Newtonian physics often treats friction in this way. The formal principles guiding exclusions of alternative causes are usually not provided.
h *Understanding the strength of a causal relation.* Even knowing that there may be many causes of a relation, attending to just two variables showing a relation tends to lead to an overestimation of the strength of the causal link between the two. This is a natural result of the limitations of human cognition: It is easier to imagine a causal connection between variables in front of us than to consider or invent other possible causes.
i *Understanding the inter-relation and covariation of the most important causes.*
j *Understanding causes of the measurement noise*: What is random noise for one researcher might be caused by factors identified as causes by another researcher.
k *Understanding second and higher order causes*: For example, what causes the differences or lack of differences when the relation of two variables for one group is compared to the relation of those variables for another group.
l Realization that the causal account is in the eye of the beholder, giving the scientist the satisfying illusion that the account is full and complete.

This listing of ways to understand is not meant to be complete, but rather to illustrate the many ways and levels at which even simple data can be understood.

### Conclusions from this Survey of Linear Regression

A pattern as seemingly simple as linear regression, something many scientists believe they understand, permits many different levels of understanding. Explaining linear regression to others would similarly permit many different levels of explanation, and the recipients of the explanation would comprehend the communication at one or more levels that would not necessarily match that of the explanation. Further, the ways of explaining would in most cases not be determined by a goal of communicating ‘complete’ or ‘deep’ understanding, but rather would depend on the recipient or audience and one’s communication goals. In addition, it is likely that explaining typically produces an even less complete understanding by the recipient than the communicator. Linear regression is just one example but strongly suggests that the issues are universal, that scientists’ reasoning is based on partial and incomplete understanding. In some cases the scientists may be well aware of those gaps, but all too often they are not.

### Implications of the Issues and Illusions of Understanding and Explanation

We aim this essay at scientists in all fields. Many of the issues we raise have been discussed at length by philosophers and historians of science but most scientists are not highly conversant with publications in those fields. Explaining the way that philosophers and historians have addressed these issues would lead to a much longer and different essay. Instead we try to explain in a way that practicing scientists and researchers developing scientific theories can relate to through their own experience.

The issues in this part of the essay are hinted at in the earlier section but are complex, overlap with one another in ways hard to define, are not clearly distinguishable, and defy easy categorization and organization. This part of the essay organizes the issues in a way we judge convenient and likely not controversial: Individual illusions, social illusions, and scientific and societal illusions. Some of these illusions of understanding had been discussed in the first part of this essay and those discussions will not be repeated.

### Illusions of Understanding Arising Within Individuals

#### Deduction, Induction, Prediction and Causation

These lie at the heart of the scientific enterprise. A discussion of these, and the fuzzy boundaries between them, even a discussion restricted to the role they play in illusions of understanding, might require books, not a section in this essay. The following brief remarks should be viewed with this in mind.

A major part of scientific understanding involves the search for the causes of observed phenomena, a matter of induction usually represented as hypotheses, models and theories. Deduction plays a role in every aspect of forming an induction: A scientist will usually consider many theories en route to reaching a favorite, using deduction to determine the predictions of each. There are normally too many theories one might consider to deduce the implications of each with precision, so the initial steps of induction tend to involve deduction by intuition followed by more formal treatments as induction proceeds. Given the many examples of intuitions that mislead, this factor alone makes clear the difficulties of induction. Even when a scientist formalizes a theory in equations or computer code, the process of deduction can be difficult to understand and verify: Equations may be too difficult to lead directly to predictions, often requiring numerical approximation that itself must be justified. Computer code can be implemented and produce output but whether it implements properly a scientist’s beliefs and hypotheses can be difficult to ascertain, and computer algorithms can be far too complex to understand.

When induction does work reasonably well, it produces a causal account that leads humans to believe they understand, often because a model predicts observed data. In the case of linear regression we saw what is often the case, that the ability to predict data is mistakenly thought to imply a causal connection. This mistake can be viewed as a confusion of deduction (correctly deducing the value of one variable given a value of another) and induction (interpreting the cause of the correlation). Lord’s two theoretical statisticians were aware that a given starting weight could be used to predict a weight at semester’s end, but were trying to infer (induce) whether girls and boys were gaining weight differentially (which then might lead to inducing ‘why’). The conclusions of Lord’s two statisticians, like all theories and models, were induced. However, it is best that a scientist should keep in mind that theories are never ‘correct’. They are meant to represent what is currently the ‘best’ account, based on everything known to the scientist. Sometimes the best account of a correlation will have one of the variables as a primary cause of the value of the other. Other times the primary cause will be quite different, the simplest case arising when a third variable is the best cause of a correlation between two other variables. To take a trivial example, one might produce a regression showing that the rate of drowning deaths increases as a function of the amount of ice cream sales, but a model positing ice cream causes drowning would not be a best model. Rather, the changes in temperature over the year would be a better account of the changes in both variables. The obviousness of this better account should not be allowed to make one think that proper interpretation of a regression is easy. In this case it is difficult to think of a reason why ice cream might be a significant cause of drowning (perhaps one might come up with a minor cause such as ‘cramping’). Thus one is led to look for another and better cause.

In other cases a direct causal account may seem plausible until one thinks of, or most likely is led to think of, a better account. For example, one might observe that the amount of aggression a male child shows in school is correlated with the number of hours playing violent video games. Here it is plausible that there is a direct causal link. The existence of a plausible causal account may give the illusion of understanding. Yet one of a host of alternative accounts might be parental supervision: Parents who limit the amount of such video game playing might better train their children to inhibit aggression, or be better models for them. One should not fool oneself into thinking these issues are easy to resolve. Lord’s paradox shows how the best methodologists in the world can reach divergent interpretations (different inductions of causes) that are each strongly held. Stein’s paradox shows that a correct mathematical deduction interpreted causally appears paradoxical (“How could unrelated phenomena affect each other?”). Simpson’s paradox shows how intuitions can go awry both for scientists and laypersons.

Possibly the most pervasive illusion of all is the belief that a ***single*** model, hypothesis, or theory specifies the cause of the phenomena under investigation. There are almost always multiple causes at different levels of complexity and depth. If there are data showing that someone has pushed open a door, there are many levels of understanding, each of which might be viewed as a valid cause. Some examples: “the desire to make use of what is in the room”; “the many cognitive factors that have combined to make the room entry desirable at that moment in time, including one’s personal history”; “understanding the neural network dynamics that have led to and enabled the door pushing”; “the understanding that the door will open if more than a minimum amount of force is applied”; “understanding the biomechanics of body, hand, and eye coordination, and the activity to the peripheral nervous system that enables and produces the push”; understanding the chemistry at the synapses that enables the neuronal firings to take place in the amounts and dynamics needed to produce the push”; “understanding the physics that allow the hand to push rather than penetrate the door”. Thus as far as we know, there is not ever a single cause, but many causes. Context in a given setting often makes us think of one cause that seems particularly compelling, sometimes in a belief that the outcome would not have occurred had the cause not been present (Pearl, 2000). However it would be a mistake to think such a cause is the only one. At the highest levels of cognitive understanding the different views of causes are often seen in legal disputes about the causes of accidents and other problems (e.g. Gill & Keil, 2022).

This discussion has utilized terms like ‘primary cause’ because the deepest cause might be the organization, structure, and rules of the universe we inhabit, and for all we know those might be infinitely complex. It can be more fruitful to consider causality a cognitive construct that will vary with context and individual. But what is judged primary by an individual will vary with their knowledge and their assessment of that knowledge. An important factor for many individuals will be counterfactual reasoning (Pearl, 2000): A factor might be judged an important cause of a given phenomenon to the degree that the individual believes the phenomenon would not have occurred if the factor had not been present. But that reasoning is itself an induction based on everything one knows and its interpretation, and will usually change when one learns new factors. A car driven by a driver with a certain level of intoxication rear ends another. Is the cause a failure to brake in time? The recent imbibing of alcohol? The death of a mother that led the drive to drink alcohol for the first time ever? The disabled brake lights on the car rear ended? The fact that the driver of the rear-ended car had on four prior occasions collected insurance payments for similar accidents? Not only does the judgment that the accident would not have happened without the presence of each of these factors vary with the knowledge that the factor was present, and vary with the individual’s assessment, but varies with the way all these factors interact. Identifying the most useful counterfactual can be exceedingly challenging (e.g. Byrne, 2024).

These ambiguities of causality infect every aspect of scientific theorizing. Scientists often infer causality from interventions. Adding or subtracting a factor from a control condition causes a change. But that is only the start of induction: What is the cause of the change is not necessarily what the scientist believes or proposes, due to what can be one, a few, or a large number of confounding factors. In fact the job of reviewers and editors of journal submissions is in good part the identification of confounding factors that provide an alternative and sometimes better causal account than the proposal in the submission.

Even the most obvious statement about causality, that a cause precedes the effect can cause confusions when inducing causes (and not just in fundamental physics, but in everyday inductions). E.g. consider Newcomb’s paradox: God predicts without error whether agent A will choose both envelopes each containing either $1000 or $1, or take just one; a prediction of both leads God to fill both with $1; a prediction of one leads God to fill both with $1000. Using God to make the predictions can confuse some people. Consider a variant in which the prediction is based on what agent A chooses in an identical prior situation without present memory of the prior choice. Choosing both will clearly tend to produce an outcome of $2, and choosing one will tend to produce an outcome of $1000, because agent A cannot help but make the same choice most of the time in an identical setting without memory. On the other hand choosing one seems to violate the direction of causality: How can the choice change what is in the envelopes that are already filled? This is a seeming paradox caused by a failure to identify the primary cause: The cause of the contents of the envelopes is not the choice made on the second occasion, but rather the way of thinking used by agent A, which tends to be the same on both occasions.

In sum, induction of a ‘best’ cause is an extremely difficult matter, often distorted by illusions of understanding that involve deduction and prediction. Most of this essay addresses this issue.

### Illusion of Understanding Due to Correlation

Scientific theorizing almost always begins with observations of data patterns. When the relation of two (or more) variables are roughly linear or have a linear component their relation is often described by a correlation. Most scientists seeing a correlation of x and y will begin a search for causes by assuming one of these causes the other (Galton, 1897; Pearson, 1897; Aldritch, 1995). A problem arises when there is some plausible reason for such a cause; too often such an account will stop a search for better causes. Prior research has suggested that when one draws a tentative conclusion that x causes y (or the reverse) the tendency to stop searching is magnified.

### Illusion of Understanding Due to Prediction

Mathematical or computer simulation models are often proposed as causes of a pattern of data. Usually after parameter values are chosen to produce a best fit the model predictions are judged successful, perhaps because they are better than those of one or more alternative models after model complexity is taken into account. Similar to the case of correlations, judgment of a good prediction tends to stop a search for a better model, and such a stopping tendency is magnified when prior research has produced support for the model.

### Illusion of Understanding Due to Plausibility

One’s ability to come up with a plausible explanation, even a partial and shallow one, can lead to an illusion of understanding considerably exceeding reality, and can often be misleading. Plausibility lies in the mind of the scientist and often arises due to intuition, rapid and incomplete analysis, incorrect generalization based on superficial analogy, or incomplete and sometimes incorrect knowledge (see Weisberg et al., 2008). We have seen this in the cases of regression, but partial understanding based on plausible explanations can lead to (likely) invalid conclusions in all domains, not just in the realm of correlations and regressions. Here is an example of a plausible sounding account that produces partial but incorrect understanding: “The sun is massive and has enormous gravitation. It should be easy to get rid of nuclear waste by sending it up on rockets and letting it fall into the sun.” The truth is the opposite: It is far easier to send nuclear waste out of the solar system than into the sun. (Siegel, 2019). There are scientific articles that explain results with verbal hypotheses. It is often the case that the predictions claimed to be produced by these are based on intuitions. Even the predictions can be explained plausibly they can be incorrect.

### Illusion of Understanding Due to a Belief in Explanatory Depth

The illusion of explanatory depth is the belief that one can explain a phenomenon in more depth (typically layers of mechanistic details) than turns out to be the case; for example, a person asked how a toilet works may be surprised when finding it difficult to do so (Rozenblit & Keil, 2002). This illusion is larger than illusions of other cognitive abilities such as estimates of how well one can recall a movie or make an international phone call. The illusion of explanatory depth may also play a role in the Dunning-Kruger effect (Kruger & Dunning, 1999), that most non-experts overestimate their abilities and skills to accomplish a wide range of tasks. Some of this effect is due to regression to the mean, as discussed in part one of this essay, but how much is a subject of debate. One factor driving this illusion may be a belief that one ‘knows’ what can be obtained easily from external sources such as search engines or questions to an AI (Fisher et al., 2015; Messeri, 2024). This illusion could be increased by beliefs in authority, that the source knows more than it does, or that the source has provided a deep explanation when the explanation is actually quite shallow. As access to information from external sources becomes ever easier, seamlessly and autonomously supplied, illusions of having deeper understandings will increase, with the potential of producing increasingly severe misunderstandings.

### Understanding in One Domain May Not Generalize

Science continually branches into narrower and narrower subdomains, each with their own levels of understanding and scientists specializing in each. In this way understanding continually increases. Yet each subdomain tends to develop its own terminology and knowledge that circulates mainly among its own scientists, limiting application to other domains and subdomains. That this is a problem is highlighted by examples of the gains for science and society when understanding in one domain is used successfully in other domains (e.g. scientific forensics used DNA fingerprinting, enabled by the prior discovery of the structure of DNA in molecular biology). Not only differing terminology, but also limited time for scientists to learn what is needed to understand other domains, poses serious challenges for generalizing knowledge and understanding. This is a problem that has not been resolved (Daniel, McConnell, Schuchard, & Peffer. (2022), and may worsen as science continues to develop.

### Illusions of Understanding Caused by Forgetting

Perhaps a scientist at university studied regression to the mean and understood it well; that is no guarantee that the scientist will see its presence in a study twenty years later when forgetting has taken place. It is quite common for a scientist to understand reasonably well the basis of the analyses and the rationale for conclusions at the time the research is submitted for publication. However, after what can be a considerable delay ranging from weeks to years, a delay that might be filled with other projects, it is natural for forgetting to occur: Details of the earlier study are often lost, and the understanding of the published research and its model is often reduced to little more than the conclusion reached.

Due to one’s belief that one understands, relying on one’s memory can lead to misleading designs of new studies, and misleading explanations of the causes to other scientists, students, and the public. Scientists sometimes deal with this problem through the use of explanatory notes, though notes may do more to buttress an illusion of understanding than the understanding held initially. Notes by definition are highly abbreviated texts that may do more to give a scientist the illusion of understanding than anything close to the level of understanding held when the research was carried out. One would hope that a publication would provide the best and deepest understanding, but even that is subject to the ability of the authors to explain well, is made difficult by demands or preferences by journal editors for shorter publications, and made even more difficult by the proliferation of journals with vastly different standards for quality of explanation (Seeber, 2024).

### Illusions of Understanding Caused by the Difficulty of Deduction

Deduction is carried out by logical and mathematical steps, and should not be a matter of debate if carried out properly. Of course intuition is often insufficient to produce correct deduction. We have seen an example in the case of Stein’s paradox. This is generally the case when models and explanations are probabilistic, as in the famous ‘Linda’ example of Kahneman and Tversky mentioned earlier. However, the difficulties of deduction occur in many other settings as well, even for simple models. For example, start with a fraction x between 0 and 1 and replace it by 3 × (1-x), and keep repeating with the prior result. One cannot predict many steps into the future what the result will be for a given starting value. This is known as deterministic chaos, and occurs in many much-more-complex situations such as those in real environments. Sensitivity to initial conditions is popularly known as the “butterfly effect”, so-called because of the title of a paper given by Edward Lorenz in 1972 to the American Association for the Advancement of Science in Washington, D.C., entitled “Predictability: Does the Flap of a Butterfly’s Wings in Brazil set off a Tornado in Texas?”. The difficulty of deduction seen in both deterministic and probabilistic chaos is one of the reasons it is difficult to determine causal attribution and induction.

The difficulty of understanding deductions are not limited to recurrent feedback systems and deterministic (or worse, dynamic and probabilistic) chaos. The difficulty also occurs in many situations where there are very long chains of logic and many mathematical steps. In many such cases, one does not try to work out predictions analytically but rather produces them with computer simulations. When such a program is made available to others in usable form, it is easy to confirm by running the program that the claimed output is what is produced. It is often extraordinarily difficult to know whether the program does what the program is claimed to do. Also, it can be extremely difficult to understand how the program produces its outputs when the program is very large, or is developed by evolutionary algorithms, or is trained on large data sets and produces very large models with enormous numbers of parameters. The recent advent of Large Language Models provides a case in point: The programmers may understand the learning rules but the uncertainty about the data that is used for training, the huge number of layers of the neural net and the billions or trillions of parameters (e.g. one early and ‘small’ LLM, GPT3, had about 175 billion parameters), and the possibility that the convergence on parameter values might be non-optimal, make understanding the basis for the program’s output essentially impossible. In sum, stating conditions clearly and with mathematical precision, or producing a computer algorithm used to simulate, can give the illusion of understanding, both for the author and anyone reading the descriptions. This illusion is due in part to mistaking ability to predict for understanding, and due in part to a trust in authorities that use those algorithms successfully (Messeri & Crockett, 2024). The difficulties of understanding deduction is exemplified in modern mathematics. A mathematics professor giving an invited lecture on current results is not surprised when only one or two mathematics professors attend the talk, because no one else can understand the content of the lecture. One also is reminded of John von Neumann, one of the intellectual giants of the 20th century, who once asserted to a struggling student, “Young man, in mathematics, you don’t understand things, you just get used to them.”

### Illusions of Understanding Caused by the Limitations of Human Cognition

We have seen the effects of the limitations of human cognition in the case of linear regression. Whatever is the basis for the relation of two variables depicted as a linear regression, human cognition is limited and cannot consider or imagine all the factors that might contribute to the relation. The natural result of a focus on two variables is an overestimation of the causal connection between them, especially when one can think of a plausible manner in which one variable can cause the other. To take just one example, suppose one is given data showing that higher drug addiction is associated with lower body weight. One might imagine that addiction changes dietary habits, plausible enough to make one think this is a strong cause. However, hearing that socioeconomic status is strongly correlated with both might lead one to downgrade the strength of the initial cause when one realizes there is a better account (e.g. Gandhi et al., 2024; more generally these authors evidence a tendency to overestimate the causal effect of whatever variable is being considered in the moment). Ignoring alternative causal accounts can also vary with the goal and task (Fernbach et al., 2010).

### Illusions Caused by Excess Trust in One’s Judgments and Reasoning

As discussed in part one, even experts have trouble making probabilistic inferences, especially when using intuition and when time prevents extended analysis (e.g. Tvesky & Kahneman, 1983). However, the previous section discusses the general difficulty of deduction in complex situations (as one can see in much of modern mathematics), so humans generally and scientists in particular ought not to trust their own reasoning excessively: Not allowing for the possibility of error in one’s reasoning can therefore produce an illusion of understanding. As just noted, this proviso applies to deduction, but applies as much if not more to induction of causes.

It is possible that scientists, statisticians, and methodologists are even more susceptible to excess trust than lay persons. This situation is the result of what can be described as ‘vagueness’ in knowledge. A good example is found in Bayesian reasoning and decision making (e.g. Savage, 1954; Edwards et al., 1963). In Bayesian inference, one calculates the likelihood of data according to some hypothesis and multiplies by the prior probability of the hypothesis. This is used for decision making in what is termed subjective expected utility (SEU): A decision is based on the product of the utility of an option times the probability of an option. However, for this to work, both utility and probability must be specified in a form that can be trusted. Unfortunately, most information humans use is ‘vague’: Whether or not humans attempt to specify values of alternatives or distributions of those values, and attempt to specify probabilities or probability distributions, they do not trust any of those specifications well enough to use the quantitative specifications. In fact, sufficient uncertainty can be shown to ensure that such specifications cannot be trusted (Shiffrin, 2022). As a result, almost all decisions made in life by humans (and scientists) are not based on quantitative comparisons (i.e. SEU) but on various sorts of qualitative comparisons (see also Johnson et al., 2020). These considerations imply that scientists should not completely trust their inferences; doing so can produce an illusion of understanding.

### Do Scientists Know When Their Understandings Are Illusions?

This question is difficult to answer, especially when the subject is complex (e.g. climate change or human cognition), or a model is complex (e.g. LLMs). The prior section points out that scientists may not realize that an understanding is an illusion when they give excess trust to their reasoning. In addition, a scientist asked whether they understand might find answering depends on many factors, such as the complexity of the situation or model. As complexity increases the scientist might take longer to reflect before responding; as the time between learning and answering increases, the more likely the scientist would admit of incomplete understanding. lt is less clear that a scientist would appreciate the limits of understanding when the subject or model appears to be simple, such as the case of linear regression covered in this essay. There are cases where a scientist forced to explain some phenomenon or model comes to realize that their understanding is limited, especially if those receiving the explanation ask for further clarification (Smith et al., 2009). However, there are sure to be many cases where a scientist explains something or uses some belief that is actually an illusion without realizing the limits of their understanding. Whether or not the scientist is aware of a limitation of understanding, the incentive structure in science promotes such a pretense: Reviewers and editors prefer and may demand strong conclusions.

### Illusions of Understanding Arising in Social Contexts

#### Illusions Due to Explanations Provided by others

There may be a general tendency to trust provided explanations, and there is surely a tendency to use the existence of a provided explanation to stop a search for other and better ones. The tendency to accept is magnified when the explanation matches initial intuition. Acceptance of an explanation also tends to require a certain degree of trust in the communicator.

#### Understanding Demands Trust

Earlier we discussed illusions caused by excess trust in one’s reasoning. Just as important is the need to trust judgments and reasoning by others, despite the chance that doing so can produce an illusion of understanding. Every field of science has witnessed increasing sophistication and complexity of analyses, procedures, and modeling techniques. These days they tend to be implemented as computer algorithms, and are often made public for other scientists to use. The algorithm developer often understands deeply, this understanding including the background assumptions that must be met, the situations in which the algorithm can be applied productively and usefully, and in general the conditions of proper use. There are exceptions such as cases where the developer understands well the rules by which an algorithm ‘learns’ but does not understand the final result (e.g. LLMs). Even when the developer understands well, the user of a publicly available algorithm typically understands much less, and cannot take the time to learn. In such cases, the user tends to accept and trust the algorithm’s output. We have seen that something as ‘simple’ as linear regression requires deep understanding for proper use. What then of the far more complex algorithms scientists use routinely? Statistical examples are seen in scientific articles giving sophisticated and complicated statistical procedures and calculations to justify statistical reliability and the conclusions of the analyses. Readers, including reviewers and editors are rarely willing to take the time and effort to deeply understand the procedure and its proper use, and instead assume the analyses have been used correctly.

Modern science cannot proceed without trusting these tools, but there are dangers in doing so. Scientists need to be aware that this is the case, and to be careful in drawing definitive conclusions. Thus, when a scientific article relies on hard to understand algorithms and procedures, the authors should support their research with analyses that are easier to understand, for example using exploratory data analysis (e.g. Tukey, 1977). This step is too often skipped. Recall that Lord wrote his two page article in 1967 to warn researchers to be careful and thoughtful when using a standard statistical approach to draw conclusions. His example was relatively simple, but his suggestion applies ever more strongly as the procedures become more sophisticated and complex.

#### When Should Results, Claims, and Procedures Be Trusted?

What is the basis for trust? Shiffrin et al. (2023) argued that one is generally forced to rely on a consensus of expert opinion when such a consensus exists (which is the case for Simpson’s paradox and Stein’s paradox, but apparently not for Lord’s paradox). Of course there are many examples in the history of science where the consensus has been wrong in detail or even in its main conclusions. To give a few examples, a meteorologist initially proposed continental drift around 1910 but was dismissed as wrong for decades (Oreskes, 1988, 1999); for many years it was the expert consensus that ulcers were caused by lifestyle (e.g. eating habits) or by genetics, or by stress caused by improper parenting, until the discovery of the bacterium *helicobactor pylori* in 1982 (Thagard, 1988). Shiffrin et al. (2023) made the case that reliance on expert consensus is required not only in science but in every domain where evidence evaluation is needed, such as attributions based on historical records, interpretations of archaeological and anthropological findings, or attribution of authorship of artistic productions. When the data are extremely difficult to analyze and interpret, such as in the case of climate change, or the role the gut biome plays in physical and mental health, then different expert opinions are common, and groups with expertise in different domains may reach different conclusions (e.g. whether low carb or low fat diets are better for health Hu, Mills, Yao, Demanelis, Eloustaz, Yancy, Kelly, He, & Bazzano, 2012). We may be forced to rely on expert consensus, but there are many dangers in doing so, such as a consensus based on experts relying on a single source. Checking the basis for a consensus is therefore desirable, but in too many cases is not pursued (Yousif et al., 2019).

The issues of trust are likely to grow ever more problematic as the use of Large Language Models increase: These models provide explanations in response to queries that are tailored to increase the questioner’s trust in the answer, often independently of the validity of the answer (Wester et al., 2024.)

#### Scientists Disagree

We have seen the effects of disagreement in the case of Lord’s paradox. There are many reasons why scientists disagree. Some are not defensible such as payments by a company to a scientist to induce support of a position contrary to current expert consensus when acceptance of the best science would harm company profits (e.g. Harris & Carey, 2008). Another dubious basis for disagreement is the tendency for many scientists to defend their results and theories at all costs in the face of new results demanding revision. History of science is littered with examples of scientists not only attempting to uphold their prior models well past their expiration date, but also attempting to suppress research showing the need for change (Delborne, 2016). However there are many valid reasons to disagree, sometimes based on differences in depth of understanding, sometimes based on difference in types of expertise, and many times because the best explanation is in debate. Particularly when a minority opinion is held by a significant proportion of experts it raises difficulties for everyone from scientists to students to laypersons, and best practices are not obvious (Shiffrin et al., 2025).

#### Communication

Scientists need to explain their research, their findings, and their models to co-authors and colleagues, to scientists in related fields, including reviewers and editors, to scientists in distant fields, to funding agencies, to science journalists, to students and trainees, to the lay public, and to politicians and leaders. In the following discussion let *comprehension* refer to how well the recipient understands the communication, and let *understanding* refer to how well the recipient understands the process itself. A communicator could be good at the former and not the latter (e.g., Armstrong, 1980; Ware & Williams, 1975). Similarly, the quality of a recipient’s comprehension may vary (Van Dijk and Kintsch 1983a, b; McNamara & Magliano, 2009), independent of whether the recipient truly understands the process being described (Griffin et al., 2019).

Many complex issues recur in understanding and explaining when communicating. Sometimes explanations are intentionally incomplete in order to foster comprehension, producing inadequate understanding. We saw that in the case of Lord’s paradox where his two page paper in 1967 had to be explained further in an even shorter paper in 1969 (not that that ended the controversy). Of course short communications containing short explanations can also be based on superficial and invalid understanding by the communicator, as is often the case in the formation of conspiracy theories (Lewandowsky et al., 2020). Incomplete, superficial, and invalid explanations also allow critics to object to what is the best current consensus of scientists. We have seen this for example in the attempts by the tobacco industry to deny evidence that smoking causes cancer (Brandt, 2012), and attempts by the energy production industry to deny the human causes of climate change (Oreskes et al., 2018). In contrast, the recipient of a communication sometimes finds it clearer and more satisfying when the communicator has at most a partial and incomplete understanding, as is probably the case for science journalists, and perhaps the case for scientists teaching courses covering subjects they do not themselves carry out.

Also unclear in any individual case is the best mixture of communication by such means as verbal statements, text, graphs and figures, and equations and computer codes. It is generally the case that interactive communication, by which the recipient can ask questions and receive clarification is superior to passive transmission, such as textbooks, classroom lectures, blogs on the web, and so on (Deslauriers, McCarty, Miller,, Callaghan, & Kestin, 2019; Freeman, Eddy, McDonough, Okoroafor, Jordt, & Wenderoth, 2014). Interactive communication certainly improves comprehension, but understanding can be limited by the degree of understanding of the communicator (occasionally the recipient learns the inadequate understanding of the communicator through questioning).

Communicators often have the illusion that the recipient understands what is being communicated, usually because they understand (or believe they understand) what they are saying. In addition, a recipient of a communication may gain some partial understanding, resulting in an illusion that their understanding matches that intended by the communicator.

#### Teaching and Training

Everything covered in this essay has clear relevance for teaching and training. For example, our discussion of the difficulties of understanding and explaining linear regression raises many questions about the proper way to teach statistics to students at various levels of education. The merits of individualized training are demonstrated by successes of the apprentice system throughout human history (think of Antonio Stradivarius and violin making in the 1700s), and operate today in the training of graduate students and postdoctoral researchers. Illusions of understanding raise many issues concerning best practices for teaching and training but this is an enormous subject in its own right, far too large for any detailed treatment in this article. We limit our remarks only to mention of the inadequacy of STEM education, especially in the United States (Burke et al., 2022), At least one cause of the failure is seldom addressed: The difficulty of deeply understanding even the simplest phenomena. It might help if students at each level of education were made aware that deep understanding is difficult even for the most expert humans, and it might help if students were asked to achieve not the deepest level but one useful for their own needs, an understanding that becomes somewhat deeper as their education proceeds.This strategy fits with research arguing that learners fare better if instructors adopt a growth mindset by which they try to help their learners improve and learn more, not necessarily learn everything or become expert (Muenks et al., 2020; Murphy and Dweck, 2024).

### Illusions of Understanding Arising in Science and Society

#### The Illusion of Accepted Knowledge

Theories of causes are not static but evolve as science progresses. It is always an illusion that the present accepted account of cause is the best and deepest. Given that the current best account is usually associated with the most respected scientists, it is by far the easiest path forward to accept the current consensus account as valid. Countering this tendency to a degree is the desire of some scientists to make a ‘name’ for themselves by showing that accepted dogma is incorrect. The difficulty with such research is the desire of the authors of accepted dogma to protect their account, sometimes to the extent of preventing publication of alternatives.

#### Partial Understanding Produces and Hinders Scientific Progress

Human progress (scientific progress in a broad sense) can be achieved with little or no understanding at a complete or deep level. This has been shown by inventors and inventions throughout human history. E.g. the ancients carved rock with only the knowledge that water expands, by using wooden wedges that expanded when wet, or heating water placed in crevices; airplanes were made to fly with little more knowledge than the passage of air over the wings were somehow responsible; today LLMs produce human discourse with little more understanding than that they have been trained on a huge number of exemplars to predict next output. Most people are interested in causes, but given that something works, many are satisfied with a superficial explanation, especially one that seems plausible. In contrast, scientists are most often interested in finding and verifying deeper causes of the phenomena they study. In both cases the various illusions of understanding discussed in this article can hinder progress. For example, an inventor can be led down the wrong path by an incomplete and superficial understanding of the causal mechanisms involved. A scientist may be operating with a better and deeper understanding but will be no less hindered by similar illusions.

Sloman and Fernbach (2017) argue that humans deal with cognitive limitations because we live in a rich community of knowledge, and constantly draw on information and expertise stored outside our heads: in our bodies, our environment, our possessions, and the community with which we interact. This is surely the case, but the knowledge we rely on is sometimes incomplete, incorrect, and varies with the sources of knowledge of an individual. The latter is increasingly a problem in these days of social media and information silos (e.g. Ivic, Rubinelli, Franco, Sams, Ginwal, Obregon, & Ratzan, 2025).

Science has become ever more complex, including its tools, making understanding ever more difficult. If linear regression is hard to understand, what should we make of even slightly more complex tools such as MANCOVA, principal component analysis, structural equation modeling, Bayesian inference and Bayesian model comparison? Faced with such difficulties, science has turned to assists from AI, but the algorithms that help us are themselves difficult if not impossible to understand. In the philosophy of mathematics, a non-surveyable proof is a mathematical proof that is considered infeasible for a human mathematician to verify and so of controversial validity (Tymoczko, 1979). These issues are important in science generally: The field of ‘explainable AI’ has mushroomed in recent years in an attempt to deal with the difficulty of understanding AI algorithms, but has not provided answers as much as identified problems.

There are many levels of partial understanding, and which level is best for different purposes is not obvious. The deepest level will generally not be useful for teaching, for communication, and for explaining to others who are not experts in one’s narrow domain of research. On the other hand, too superficial an understanding can lead to serious problems as can be seen for example in numerous examples of ‘unintended consequences’ (e.g. in Australia, European rabbits introduced for hunting and cane toads introduced to control beetles proliferated causing immense problems; placing tariffs on imports in order to protect domestic industries and jobs from foreign competition, can end up costing more jobs than are saved by protectionism (Dinopoulos Elias, Heins, Gunnar, Unel & Bulent, 2024).

#### Increasing Accuracy of Partial Understanding

The natural evolution of science has seen a narrowing of fields of inquiry coupled with better and better accounts of phenomena in those fields. To the degree that the explanations do not generalize across these fields (see for example Goldstone et al., 2010), it could be said that we are learning more and more about less and less (possibly a problem or a natural progression); but important exceptions occur when science uncovers new general principles that apply across fields and domains. For example, light was once considered an entity on its own but is now understood as one slice of a much larger spectrum of electromagnetic radiation. Sometimes intentionally simplified ‘ideal’ models are employed to aid understanding, provide approximate predictions, and indicate promising directions for new theory (e.g. Shiffrin and Steyvers (1997), produced a good model for recognition memory using Bayesian reasoning to make optimal predictions based on radically simplified assumptions, and that model produced good approximations.) Other times quite incomplete abstractions produce useful understandings that are applicable in many settings, as when good decision making is helped by use of fast and frugal heuristics (Gigerenzer & Todd, 1999; e.g. German students asked whether San Antonio has a larger population than San Diego are much better than American students because they use the heuristic “guess the city you have heard of”).

#### Partial Understanding and Exploitation Versus Exploration

Progress in science can leap forward when unanticipated results are found that are difficult or impossible to explain with current understanding (e.g. Kuhn, 1962). When such results are found and reported, it is no easy matter for the field to determine whether the results are valid, or due to error, statistical noise, or experimenter bias. There is a natural tendency for the field to take the position that unexpected findings and novel theories are incorrect. Here we are discussing biases caused by beliefs, but such beliefs can have real effects on the mind and body, such as those seen in the use of placebos (Collaca & Miller, 2011). Thus, strong biases produced by belief are a natural outcome of its real effects.

A belief that one’s theories are correct or mostly correct can produce a focus on research designed to confirm current belief, or on research designed to produce minor refinements (research on confirmation bias is an example – see Klayman, 1995). This could be described as research designed to exploit current belief rather than explore new avenues. An argument can also be made that attempts to deal with the so-called ‘crisis of reproducibility’ lean too far in the direction of attempting to uphold current beliefs (e.g. by making replication a primary goal of science, and by imposing stricter statistical standards that might hinder publication of new results and theories).

On the other hand, partial and incomplete understanding can lead a scientist to design new studies incorrectly when trying to verify current beliefs, and thereby produce unanticipated new results that move science forward. In fact a case can be made that certain types of ‘random’ experimentation can benefit science more than designed experimentation (Dubova et al., 2022; Musslick, Hewson, Andrew, Strittmatter, Williams, Dang, Dubova, & Holland 2023; Dubova et al., 2025).

#### Knowledge Acquisition and Maintenance

For science to proceed effectively, it is not enough to understand well and deeply: Science is a collaboration among many researchers, some communicating directly, in person or through internet based channels or telephone, and others indirectly through publications and other writings. In recent years the number of journal articles, archived articles, blogs, and books has exploded (e.g. in 2022, entries in the Scopus database showed that total worldwide S&E publication output reached 3.3 million articles). Such numbers make it impossible to find what is currently relevant by reading what is available. One might think it possible to peruse titles and abstracts, or rely on increasingly sophisticated search engines, but the problems of understanding we have been discussing make this problematic: The domain of research indicated by a title is often misleading, due to the ‘fuzziness’ of language and different uses of terminology in even slightly different fields and even in the same field by different authors. Most critically, the claims made in abstracts (or in conclusions at an articles end) cannot be taken at face value. Evaluating the claims, either about the effects found or the implications of those effects, requires a deep level of understanding, possibly even deeper than that held by the authors. Even if the authors did have a sufficiently deep understanding to make the claims reasonably valid, the difficulties of explaining their understanding in what is often limited journal space, are legion. The authors may be less than expert in communicating, but more fundamentally may be forced to use incomplete and partial explanations and analogies that could be misleading and subject to alternative interpretations. These difficulties are not restricted to complex phenomena such as the way that aircraft wings support flight, but phenomena so simple they are described with linear regression.

These difficulties make it essentially impossible for a scientist to take the time to read each potentially relevant report in the depth required to understand and validate the conclusions. Increasingly, then, scientists rely on the social network of science, on the various forms of in person communication in conferences and workshops, or directly in face to face interactions, thereby learning what is thought to be valid and worthwhile by scientists believed to be experts.

## Outlook

Scientists use and deal with partial and incomplete levels of understanding in every aspect of their practice: domain of study; experimental design; data analysis; interpretation; model formation; journal submission; response to reviews; discussions with colleagues, lectures, teaching, training students, and much more. Their understanding typically waxes and wanes, rising from initial experiment conception through submission and sometimes revision, then waning subsequently as forgetting takes place, and then perhaps changing significantly when new results demand new theories and new conceptions.

The degree to which individual scientists are aware of this situation is unclear, but the lay public is far less aware, likely due to the way scientific results and theories are publicized. Especially in recent years we have seen certain members of the public, including some policy makers and politicians, distrusting science. But this distrust is in most cases not based on awareness of scientists’ difficulties of understanding, but rather on dislike of what scientific consensus implies for proper action in society. Conversely many members of the public do trust science but do so while assuming scientists do not have illusions of understanding.

Advances in technology and complex quantitative approaches, and recent advances in AI such as large language models may give many people an illusion that we are gaining more and deeper understanding than we are, and that there are now relatively few things left for science to learn. Such beliefs may be fostered not just because people see the advances in public facing interfaces (e.g. ChatGPT) but because the advances have a veneer of formality and logic. But the reality is quite different because the advances challenge the limits of human ability to comprehend them. As science has become ever more technical, scientists increasingly rely on computer programs produced by others, again without deeply understanding how they work and hence not knowing their proper use. Neither scientists nor the public have much choice in this matter: Neither time nor availability of ‘teachers’ would allow one to gain the understanding of such systems. The fact that science has progressed as well as it has, as seen for example in the accelerating progress in certain fields of biology, suggests that our reliance on algorithms we may not understand very well is reasonable, and perhaps necessary and the most we can expect.

At the least, the difficulties of understanding should make all scientists both skeptical and humble, knowing that ‘truth’ is an ever receding goal. We must realize the full range of possible illusions and appreciate that they occur in all approaches. We can reduce false insights and achieve better understanding by embracing diverse theoretical perspectives, research methods, and modes of analysis. This requires constant intellectual humility towards our own theories, models and methods. This recommendation unfortunately competes with forces in scientific societies that foster overstated claims and conclusions: Faced with millions of publications each year, scientists must fight for attention, readers and citations in the research marketplace, and journal editors must seek impact factors, submissions, and readers. In addition, scientists’ beliefs in overstated claims are subject to biases that occur more broadly. Thus, scientists can exhibit trust that increases beyond what is justified for mentors, friends, colleagues, same institutions and affiliations, famous scientists at leading universities and research centers, and decreases for women and minority authors. Do scientists realize the extent to which such factors operate and are they adapting to their existence?

## Data Availability

Not applicable.

## Footnotes

1 Philosophers distinguish induction and abduction; we will use the more common term induction to refer to both.

2 Although the data here are hypothetical and meant to represent an ideal situation in which massive amounts of data had been collected, the situation occurs often in real life, and in fact the figure for each of the girl and boys has the same structure as Galton described for parents’ and their childrens’ heights.
